# Bioactivity‐Guided Isolation of β‐Sitosterol From *Terminalia glabrescens* as a Potent Anti‐Zika Virus Agent

**DOI:** 10.1002/cbdv.202502860

**Published:** 2026-01-14

**Authors:** Rosângela Santos Pereira, Priscilla Rodrigues Valadares Campana, Vivian Vasconcelos Costa, Vinícius Gonçalves Maltarollo, Rodrigo Maia de Pádua, José Hugo Sousa de Gomes, Grasiely Farias de Sousa, José Dias de Souza Filho, Daniele da Glória de Souza, Mauro Martins Teixeira, Fernão Castro Braga

**Affiliations:** ^1^ Department of Pharmaceutical Products, Faculty of Pharmacy Universidade Federal de Minas Gerais Belo Horizonte Brazil; ^2^ Department of Biochemistry and Immunology, Institute of Biological Sciences Universidade Federal de Minas Gerais Belo Horizonte Brazil; ^3^ Department of Chemistry, Institute of Exact Sciences Universidade Federal De Minas Gerais Belo Horizonte Brazil; ^4^ Department of Microbiology, Institute of Biological Sciences Universidade Federal de Minas Gerais Belo Horizonte Brazil

**Keywords:** β‐sitosterol, in silico similarity analysis, SH‐SY5Y cells, *Terminalia glabrescens*, Zika virus

## Abstract

Zika virus (ZIKV) infection remains a global health concern due to its neurological complications and the lack of specific drugs or vaccines. This study investigated the anti‐ZIKV potential of an ethanolic extract from *Terminalia glabrescens* leaves. Dereplication of the extract by UPLC–ESI–MS/MS identified 27 phenolic compounds, while GC–MS analysis of the dichloromethane (DCM) fraction revealed carboxylic acids, esters, an alcohol, a triterpene, and hydrocarbons. The extract (30 µg/mL) reduced viral load by 2.6 log in Vero CCL‐81 cells and inhibited viral replication in SH‐SY5Y cells (CC_50_ = 130.2 ± 27.5 µg/mL; EC_50_ = 20.4 ± 10.2 µg/mL; SI = 6.4). Bioguided fractionation localized the antiviral activity to the DCM fraction, which reduced viral load by 5.1 log in Vero CCL‐81 and 4.0 log in SH‐SY5Y cells. Chromatographic fractionation of DCM fraction afforded glutinol, β‐sitosterol, and a mixture of α/β‐amyrin. Among these, β‐sitosterol exhibited the most significant activity against ZIKV in SH‐SY5Y cells (CC_50_ > 300 µM; EC_50_ = 71.3 ± 7.1 µM; SI > 4.2). A 2D similarity analysis based on structural fingerprints revealed that β‐sitosterol is structurally distinct from known anti‐ZIKV compounds in the ChEMBL database, underscoring its novelty and potential as a lead scaffold for antiviral drug development. These findings identify β‐sitosterol as a promising candidate for the development of anti‐ZIKV drugs.

## Graphical abstract

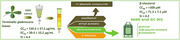

## 1 Introduction

Zika virus (ZIKV), a member of the Flavivirus genus, remains a public health concern in several countries. Since the 2015 outbreak in Brazil [1], ZIKV has drawn global attention due to its association with microcephaly, congenital Zika syndrome (CZS), Guillain–Barré syndrome, and other neurological disorders [2, 3]. Although the epidemic has subsided, Brazil continues to report ZIKV and CZS cases. Between Epidemiological Weeks 1 and 22 of 2025, 3601 probable Zika cases were registered in Brazil, corresponding to an incidence of 1.8 cases per 100 000 inhabitants, which represents a 7.2% decrease compared with the same period in 2024 [4]. Currently, no vaccine or specific antiviral treatment is available, and clinical management remains supportive.

Several classes of natural products, including polyphenols, diterpenes, triterpenes, steroids, and alkaloids, have been reported to exhibit anti‐ZIKV activity [5, 6, 7, 8, 9, 10, 11, 12, 13, 14]. Among these, triterpenes and polyphenols appear the most promising. A previous screening of 36 plant extracts rich in these compounds for ZIKV inhibition led to the identification *Terminalia phaeocarpa* Eichler, *Maytenus rigida* Mart., and *Echinodorus grandiflorus* Micheli as the most active [10].

To further explore the anti‐ZIKV potential of the above‐mentioned natural product classes, we selected *Terminalia glabrescens* Mart. (Combretaceae) for investigation, as it contains polyphenols, triterpenes, and steroids [15]. The species is widely distributed in Brazil, being popularly known as *capitão* [15]. *T. glabrescens* is traditionally used in the country to treat intestinal infections, stomach ulcers, and intestinal colic, among other uses [16].

We herein report a bio‐guided phytochemical investigation of an ethanolic extract from *T. glabrescens* leaves evaluated against ZIKV‐infected Vero CCL‐81 and SH‐SY5Y cells, which led to the isolation of β‐sitosterol as the active compound. In addition, a two‐dimensional (2D) structural similarity analysis based on molecular fingerprints was performed to compare β‐sitosterol with known anti‐ZIKV compounds in the ChEMBL database. This study provides the first evidence of the anti‐ZIKV activity of *T. glabrescens* and its bioactive constituent, β‐sitosterol.

## 2 Experimental Section

### 2.1 Phytochemical Study

#### 2.1.1 Plant Material

The leaves of *T. glabrescens* were collected at the Ecological Station on the *campus* of the Universidade Federal de Minas Gerais (UFMG) in Belo Horizonte, Minas Gerais, Brazil (19°52′37.828″ S 763°58′27.363″ W) in January 2017. The species was identified by Prof. João Renato Stehmann, from the Botany Department, Institute of Biological Sciences (ICB), UFMG. A voucher specimen (BHCB201961) is deposited in the Herbarium of the Botany Department BHCB, UFMG. The collection and research with the plant material was duly recorded with the Conselho de Gestão do Patrimônio Genético (CGEN/SISGEN), under the registration number AC53C04.

#### 2.1.2 Extract Preparation

The leaves of *T. glabrescens* were dried in a ventilated oven at 40°C, with forced air circulation. The dried leaves were milled in a knife mill, and the powdered material (300 g) was extracted by exhaustive percolation with 96°GL ethanol at room temperature. The solvent was removed in a rotary evaporator under reduced pressure at 40°C and the residue was let in a desiccator to eliminate the residual solvent to afford 84.3 g of the extract.

#### 2.1.3 Extract Fractionation and Isolation of Compounds

A portion of the extract (50 g) was dissolved in 70 mL of methanol and incorporated in silica gel (22.5 g). After solvent evaporation, the material was deposited on the top of a silica gel flash column (250–400 mesh; 150 g; 50 × 5.5 cm i.d.) and sequentially eluted with *n*‐hexane (2 L), dichloromethane (DCM) (3 L), ethyl acetate (EtOAc) (1 L), and methanol (1 L). The solvents were removed by evaporation on a rotary evaporator under reduced pressure at 40°C to afford the *n*‐hexane (HEX, 80.4 mg), DCM (1206.4 mg); EtOAc (1189.0 mg), and methanol (MeOH, 38590.0 mg) fractions. A portion (750 mg) of the DCM fraction was subjected to silica gel flash column chromatography (70 g; 52 × 1.8 cm i.d.), using a gradient of *n*‐hexane (*n*‐Hex)/EtOAc for elution to afford 281 fractions of 12 mL each. The fractions were pooled into 38 groups according to the similarity of their thin layer chromatography (TLC) profile, using sulfuric anisaldehyde as spray reagent, to afford compound **1** (12.3 mg; eluted with *n*‐Hex/EtOAc 96.5:3.5), **2** (28.1 mg; eluted with *n*‐Hex/EtOAc 90:10), and **3** (11.1 mg; eluted with *n*‐Hex/EtOAc 85:15). Analysis of the grouped fractions also revealed the grease solids **4** (176.2 mg), **5** (16.4 mg), and **6** (15.1 mg) eluted with *n*‐Hex/EtOAc (98:2), **7** (24.5 mg; eluted with *n*‐Hex/EtOAc 95:5), **8** (2.6 mg; eluted with *n*‐Hex/EtOAc 92.5:7.5), **9** (5.0 mg), and **10** (2.7 mg) eluted with *n*‐Hex/EtOAc 90:10) (Table 1).

**TABLE 1:** Compounds obtained from the fraction of the DCM fraction by flash chromatography over silica gel column.

Compound	Yield		*n*‐Hex/EtOAc (v/v)
	(mg)	(%)	
**1**	12.3	1.64	96.5:3.5
**2**	28.1	3.75	90:10
**3**	11.1	1.48	85:15
**4**	176.2	23.49	98:2
**5**	16.4	2.19	98:2
**6**	15.1	2.01	98:2
**7**	24.5	3.27	95:5
**8**	2.6	0.35	92.5:7.5
**9**	5.0	0.67	90:10
**10**	2.7	0.36	90:10
Recovery	294.0	39.20	

#### 2.1.4 UPLC–DAD–ESI–MS/MS Analysis

The analyses were conducted using a Waters Acquity UPLC system (Waters, USA) equipped with a binary pump, autosampler, degasser line, and photodiode array detector (DAD), coupled to an Xeco Triple Quadrupole MS detector (Waters). The UV spectra of the compounds were recorded over a wavelength range of 190–500 nm. Mass spectra were acquired using a triple quadrupole detector with an electrospray ionization (ESI) source, operating in both negative and positive ionization modes. The putative identification of constituents in the extract and fractions was achieved through multiple reaction monitoring (MRM) analysis, following the method described by Singh et al. [17], with modifications Supplementary material.

The analysis utilized an ACQUITY UPLC HSS C18 column (2.1 × 50 mm i.d., 1.7 µm; Waters) maintained at 40°C. Elution was carried out with water (A) and acetonitrile (B), both containing 0.1% v/v formic acid, at a flow rate of 0.3 mL/min, using the following gradient: 5%–10% B over 12 min, 10%–20% B over 8 min, 20%–25% B over 5 min, 25%–30% B over 3 min, 30%–35% B over 4 min, 35%–40% B over 13 min, and 40%–95% B over 5 min, followed by an isocratic elution step at 95% B for 5 min. The system then returned to the initial conditions (5% B) within 1 min. A 4‐min re‐equilibration period was implemented between consecutive chromatographic runs. The capillary voltage was set to 4500 V, and the cone gas voltage was either optimized or ramped, depending on the compound being analyzed (refer to Table 2).

**TABLE 2:** Compounds identified putatively by UPLC–DAD–ESI–MS/MS in the ethanol extract (EE) of *Terminalia glabrescens* leaves and derived ethyl acetate (EtOAc) and methanol (MeOH) fractions.

Compound	RT (min)	[M−H]^−^ (Da)	MS > MS (Da)	Collision energy (V); mass range (*m*/*z*)	EE	EtOAc	MeOH
3‐(4‐Hydroxyphenyl)propionic acid	0.69	165.1	121	Ramp (5–17); 70–200	×	—	—
Ellagic acid	17.49	301.1	257, 229	Ramp (5–50); 70–630	×	×	×
Gallic acid	0.90	169.1	124.9, 78.9	50; 70–200	×	×	×
Genistic acid	1.66	152.9	109.3	Ramp (10–30); 70–160	×	×	×
Homovanillic acid	0.57	181	136.7	Ramp (5–17); 70–280	×	×	—
*p*‐Hydroxybenzoic acid	1.85	136.9	93.0	18; 70–280	×	×	—
Protocatechuic acid	1.65	153.1	109, 108	16; 70–160	×	×	×
Quinic acid	0.58	191	127, 85	40; 70–200	×	×	×
Astragalin	19.00	447.1	300, 284	15; 100–450	×	×	—
Kaempferol	21.14	285	257, 229	Ramp (5–27); 70–300	×	×	—
Catechin/epicatechin	3.79	289	245.1, 205 203, 179, 137, 125	Ramp (5–27); 70–300	×	×	×
Corilagin	4.67	633	301, 249	Ramp (5–59); 70–660	×	×	×
Galloyl‐hexoside	0.81	331	271, 211, 169	20; 70–365	×	×	×
Galloyl‐HHDP‐hexoside	2.67	633.0	301, 275	Ramp (5–59); 70–650	×	×	×
Hesperidin	17.36	609	301	Ramp (5–56); 100–620	×	×	×
Hexahydroxydiphenol (HHDP) hexoside	0.59	481	301, 275	(6–40); 100–510	×	×	×
Hyperoside	17.32	463.1	300	Ramp (6–40); 100–470	×	×	×
Isorhamnetin	19.98	315	300	Ramp (5–33); 70–365	×	×	—
Myricetin	20.76	317.2	151	Ramp (5–29); 100–365	×	×	—
Naringenin	22.27	271.1	151.1	Ramp (5–27); 70–300	×	×	—
Dimeric procyanidin B2/B1	3.64	577	407, 289	Ramp (6–50); 100–600	×	—	×
Punicalagin	4.96	1083	1030, 781, 601, 575	Ramp (6–100); 100–1090	×	—	×
Punicalin	4.65	781	601, 451, 300	Ramp (6–74); 100–800	×	—	×
Quercetin‐*O*‐hexoside	19.08	447.4	301, 179, 151	Ramp (6–40); 100–450	×	×	×
Rutin	17.43	609	300, 301	Ramp (5–56); 100–620	×	×	×
Terflavin B	8.49	783.0	759, 451	Ramp (6–74); 100–800	×	—	×
Vitexin	17.13	431	341, 311, 283, 269, 120	Ramp (5–42); 70–450	×	×	×

Abbreviations: [M−H]^−^ (Da), deprotonated ion in Daltons; ×, identified; —, not identified; RT, retention time.

#### 2.1.5 GC–MS Analysis

The GC–MS analyses were performed in a GC‐MS‐QP2010 ULTRA chromatographic system (Shimadzu, Japan) equipped with NST‐05 capillary column (Restek; 30 m × 0.25 mm i.d.) composed of 0.25 µm dimethylpolysiloxane (95%) and diphenyl (5%) as stationary phase. The injector temperature was set at 250°C, the split ratio of injections was 1:50 and 1:100, and the helium gas flow rate was 0.71 mL/min. The column was initially set at 50°C and a linear temperature ramping was applied at a rate of 3°C/min until reaching 280°C, which was sustained for 25 min. The sample injection volume was 1.0 µL. The interface and detector ion source temperature (electron impact at 70 eV) were set to 250°C. The obtained spectra were compared with the spectra libraries NIST‐14 and NIST‐27 (Special Database), flavors and fragrances of natural and synthetic compounds (FFNSC), and Wiley Library of LabSolution/CGMS Solution software (version 4.20). The threshold for determining compound identity based on the similarity index between the experimental spectrum and the library was established at 85%. Samples were subjected to sialylation using N‐methyl‐*N*‐(trimethylsilyl) trifluoroacetamide (MSTFA) prior to being analyzed by GC–MS.

#### 2.1.6 NMR Analysis

The NMR spectra were recorded on a Bruker NMR spectrometer Ascend 600 MHz model, at the Chemistry Department, UFMG. The analyses were performed at 25°C, employing tetramethylsilane (TMS) as internal standard for both nuclei. Deuterated chloroform (CDCl_3_) was used for solubilization of the samples. The chemical shifts (*δ*) were recorded in ppm and the coupling constants (*J*) are given in Hz.

### 2.2 In Vitro Anti‐ZIKV Activity

Vero cells (ATCC CCL‐81; Research Resource Identifier (RRID) CVCL_0059), SH‐SY5Y cells (ATCC CRL‐2266; RRID CVCL_0019), and a clinical isolate of ZIKV (strain HS‐2015‐BA‐01; GenBank accession number KX520666) were used in the in vitro anti‐ZIKV assays. The stock solutions of *T. glabrescens* extract and derived fractions were prepared at 20 mg/mL, whereas 500 mM concentration was adopted for isolated compounds, diluted in DMSO. To obtain the working solutions for testing, each stock solution was diluted with RPMI medium for Vero cells or with DMEM/F‐12 medium for SH‐SY5Y cells. The test solutions contained 0.3% v/v DMSO. Initially, the viability of the cells in the presence of the test sample was evaluated to determine the non‐cytotoxic concentration. Cytotoxicity of the samples was assayed in Vero CCL‐81 cells using the MTT [3‐(4,5‐dimethyl‐thiazol‐2‐yl)‐2,5‐diphenyltetrazolium bromide] method [10]. Samples that demonstrated a cell viability over 80% were selected for anti‐ZIKV testing. Cytotoxicity was also assessed using the lactate dehydrogenase (LDH) method following the protocol by Kumar et al. [18]. Antiviral activity, half‐maximal cytotoxic concentration (CC_50_), half‐maximal effective concentration (EC_50_), and selectivity index (SI) were performed as described by Pereira et al. [10]. DMSO (0.3% v/v solution) and AH‐D (100 µM solution in DMSO 0.3% v/v solution) were employed, respectively, as the negative and the positive controls. This peptide was kindly provided by Prof. Nanjoom Cho from Nanyang Technological University, Singapore. The antiviral assays were conducted in three independent biological replicates, each performed in triplicate (*n* = 9).

### 2.3 Statistical Analysis

All results are expressed as mean ± standard error of the mean (SEM). Statistical analyses were performed by one‐way ANOVA with multiple comparisons followed by Tukey's post test (ZIKV + treated versus ZIKV + DMSO). Differences were considered statistically significant at *p* < 0.05. The CC_50_ and EC_50_ values were calculated by nonlinear regression. Data were tabulated and analyzed using GraphPad Prism version 5 (GraphPad Software, San Diego, CA).

### 2.4 Chemoinformatics Profiling of β‐Sitosterol

The ChEMBL database [19] was initially used to select compounds described in literature to have anti‐ZIKV activity experimentally determined. The following keywords were employed in the search: “Zika virus,” “Zika virus NS5,” “Zika virus NS1,” “Zika virus NS2B/NS3 protease,” and “Zika virus NS3,” with outcomes only for the first two. A total of 234 compounds was retrieved from the ChEMBL database with antiviral activity at concentrations below 50 µM, considered then active compounds. In the sequence, the structure of β‐sitosterol underwent assessment within the KNIME Analytics platform [20] and vROCS software [21]. This evaluation encompassed the calculation of the 2D similarity as well as 3D similarity comprising the molecular shape (spatial conformation) and functional groups inherent to the molecules (namely color). This first analysis was carried out using different fingerprint sets: Morgan, AtomPair, RDkit, StandardCDK, and Pubchem fingerprints calculated with default options. Tanimoto coefficients were calculated to measure the 2D similarity of the compound identified in the current work with those retrieved from ChEMBL using all mentioned fingerprints. In addition, the molecules underwent a 3D conformational analysis using the OMEGA 2.5.1 software [22], designed to calculate the lowest‐energy conformer for each compound. This analysis was followed by the calculation of the most probable ionization state using fix pka with a pH of 7.4, employing the QUACPAC software (QUACPAC 2.2.2.0) [23].

In addition to the above, the assessment of 3D chemical similarity mapping was evaluated via the Tanimoto coefficient (Tc) for shape and color (pharmacophoric features represented by the position of functional groups), as well as the combo score (sum of Tanimoto coefficient from shape and color calculations, in this work normalized from 0 to 1) obtained by the ROCS 3.2.1.4 software [21]. This approach was aligned with the methodology as outlined by Serafim et al. [24] and de Almeida Marques et al. [25]. All similarities coefficients were analyzed and the values ranged from 0 (completely dissimilar compounds) to 1 (identical compounds).

In this sense, this strategy relies on the property that structurally similar compounds share biological profile. Therefore, if the compounds from ChEMBL similar to most promising compound identified in this work has a mechanism of action experimentally determined, both could share the same mechanism to be investigated.

## 3 Results and Discussion

### 3.1 Chemical Characterization of *T. glabrescens* Constituents by UPLC–DAD–ESI–MS/MS

The ethanol extract of *T. glabrescens* leaves (EE) along with its EtOAc and methanol (MeOH) fractions were analyzed by UPLC–DAD–ESI–MS/MS aiming to identify constituents previously described to occur in other *Terminalia* species, especially polyphenols. Compounds were identified based on their MS/MS fragmentation pattern and by comparison with literature data [26, 27, 28, 29]. The ion‐*daughter* transitions were analyzed using MRM, focusing on those constituents previously reported for *Terminalia* species. This approach resulted in the identification of 27 phenolic compounds in the extract, of which 22 and 19 were, respectively, found in EtOAc and MeOH fractions (Table 2).

Previous phytochemical investigation of an ethanol extract from *T. glabrescens* leaves afforded a new oleanane‐type triterpene, along with known triterpenes, flavonoids, steroids, and *n*‐alkanes [15]. Therefore, the compounds herein identified to occur in *T. glabrescens* leaves are reported for the first time. However, it is worth noting that the majority of these compounds have previously been documented as constituents of other *Terminalia* species, including *T. arjuna*, *T. chebula*, *T. bellerica*, *T. catappa*, *T. elliptica*, *T. paniculata*, *T. muelleri*, *T. ivoiriensis*, *T. sericea*, *T. parviflora*, *T. triflora*, *T. horrida*, *T. calamansanai*, *T. macroptera*, *T. citrina*, *T. ferdinandiana*, *T. brownie*, *T. argentea*, *T. oblongata*, *T. nigrovenulosa*, *T. superba*, *T. pallida*, and *T. elliptica* [17, 27, 28, 30, 31, 32]. Flavonoids (narigenin, kaempferol, catechin/epicatechin, isorhamnetin, myricetin, galoyl hexoside, vitexin, astragalin, quercetin‐*O*‐hexoside, hyperoside, hexahydroxydiphenol [HHDP] hexoside, procyanidin B2/B1 dimer, rutin, hesperidin and galloyl‐HHDP‐hexoside) constitute the main group of the identified metabolites, followed by phenolic acids (*p*‐hydroxybenzoic acid, protocatechuic acid, gentisic acid, gallic acid, 3‐(4‐hydroxyphenyl) propionic acid, homovanillic acid, quinic acid and ellagic acid) and ellagitannins (corilagin, punicalin, terflavin b and punicalagin). Six out of 19 compounds described in *T. argentea* (gallic acid, quinic acid, ellagic acid, quercetin rhamnoside, corilagin, and punicalin) [33] were also identified in the present work as constituents of *T. glabrescens*. Of note, *p*‐hydroxybenzoic, protocatechuic, gallic, quinic and ellagic acids, along with myricetin, galloyl hexoside, astragalin, quercetin rhamnoside, corilagin, punicalin, and terflavin B, previously described as constituents of *T. phaeocarpa* [34], were also identified in *T. glabrescens*. These findings corroborate the similarity of the chemical composition across *Terminalia* species and highlight the genus as reservoir of polyphenolic compounds, including hydrolysable tannins.

### 3.2 Phytochemical Investigation of DCM Fraction

In view of its anti‐ZIKV activity (see Section 3.3), the DCM fraction was selected for phytochemical investigation. Fractionation of the DCM fraction by flash column chromatography over silica gel yielded 38 grouped subfractions, some of which exhibited a greasy appearance and underwent GC–MS analysis. Compound identification was accomplished by comparing their retention indices and mass spectra with data accessible in NIST WebBook, FFNSC, and Wiley library, with a similarity threshold of > 85% adopted for compound matching. The analyses revealed similar chemical profiles across the fractions, leading to the identification of 10 compounds, including two carboxylic acids (hexadecanoic acid and octadecanoic acid), four esters (ethyl hexadecanoate, ethyl linolate, ethyl octadecanoate, and ethyl *Z*‐9‐octadecenoate), one alcohol (1‐dodecanol), one triterpene (friedelan‐3‐one), and two hydrocarbons (hexacosane and pentatriacontane).

The presence of hexadecanoic acid, octadecanoic acid, ethyl linolate, ethyl octadecanoate, ethyl *Z*‐9‐octadecenoate, 1‐dodecanol, pentatriacontane, and hexacosane in a *Terminalia* species is herein documented for the first time. Moreover, while ethyl hexadecanoate had previously been identified in *T. arjuna* [35], its occurrence in *T. glabrescens* is novel. Conversely, friedelan‐3‐one had been previously documented as a constituent of *T. glabrescens* [15].

In addition to the aforementioned greasy fractions, the chromatographic fractionation of the DCM fraction yielded three solids (C1, C2, and C3), whose chemical structures were identified by extensive NMR (1D and 2D) and MS analyses. C1 was obtained as a white solid (MP = 164°C–167°C). Its MS spectrum, recorded in the positive ionization mode, exhibited the base peak at *m*/*z* 409 [M+H−H_2_O]^+^, consistent with the molecular formula C_30_H_50_O and a molecular mass of 426.7 g/mol.

**FIGURE 1:**
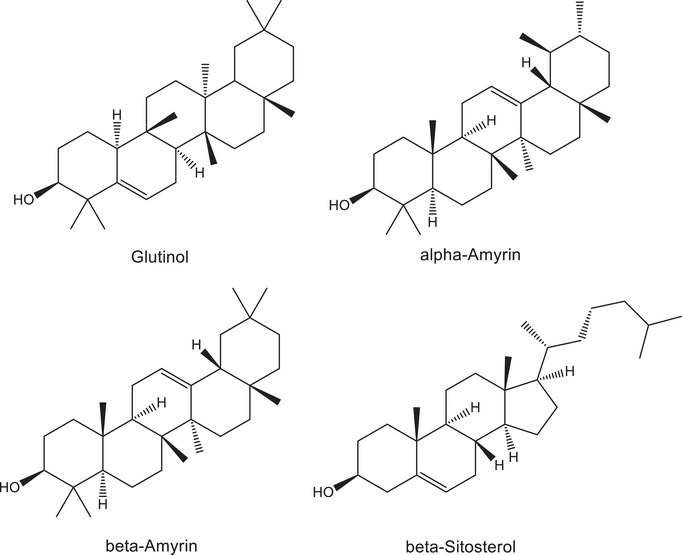
Chemical structures of compounds isolated from the DCM fraction: C1—glutinol; C2—mixture of α‐ and β‐amyrin; and C3—β‐sitosterol.

The ^13^C NMR spectrum of C1 presented 30 resonance signals, consistent with a triterpene structure. Extensive analysis of 1D and 2D NMR spectra of C1 and comparison with literature data [36] allowed its unambiguous identification as glutinol (Figure 1). The occurrence of glutinol in *T. glabrescens* is here reported for the first time.

C2 was obtained as a white solid. Analysis of MS and NMR spectra of C2 and comparison with literature records led to the identification of a mixture of α‐ and β‐amyrin (Figure 1). The occurrence of α‐ or β‐amyrin has been reported to other species of *Terminalia*, such as *T. brasiliensis* [37], *T. arjuna* [38], *T. avicennioides* [39], and *T. glaucescens* [40]. However, the presence of these triterpenes in *T. glabrescens* is novel. C3, likewise isolated as a white solid, was identified as β‐sitosterol through MS and NMR data analysis, corroborated by comparison with literature records (Figure 1). This steroid has been previously isolated from the ethanol extract of *T. glabrescens* trunk bark [15].

#### 3.2.1 Anti‐ZIKV Activity

Before conducting in vitro testing for anti‐ZIKV activity of *T. glabrescens* extract, fractions, and isolated compounds, their cytotoxicity was assessed using the MTT and LDH assays on Vero CCL‐81 and SH‐SY5Y cells. The LDH assay served to validate the findings from the MTT test and eliminate any potential false results. It was observed that the extract, fractions, and isolated compounds did not induce cytotoxic effects at concentrations up to 30 µg/mL and/or 30 µM in both cell lines (data not shown). The multiplicity of infection (MOI) for ZIKV was set at 1 and 0.001 for assays on Vero CCL‐81 and SH‐SY5Y cells, respectively, as previously established by Pereira et al. [10].

**FIGURE 2:**
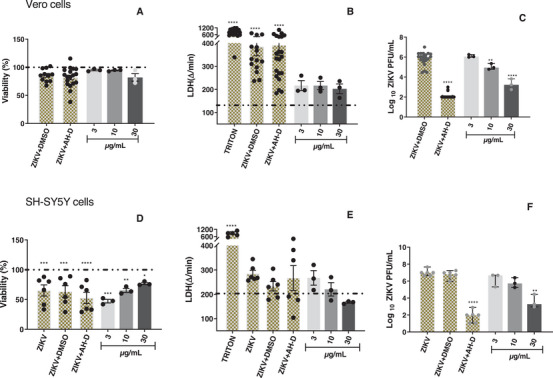
Effect of *Terminalia glabrescens* extract on viral loads and cell viability in ZIKV‐infected Vero CCL‐81 and SH‐SY5Y cells. Cell viability was assessed by MTT (A, D) and LDH (B, E) assays. Bars represent mean values ± SEM (A, B, D, E), where *p < 0.05, **p < 0.01, ***p < 0.001, and ****p < 0.0001 versus the cell control group. Triton was used as the positive control for LDH. The dashed line (A, B, D, E) indicates the cell control (without extract and ZIKV). Viral load was determined by plaque assay (C, F), and data are expressed as log plaque‐forming units per milliliter (PFU/mL). Bars represent median values, and error bars indicate the 95% confidence intervals (C, F). Statistical analyses were performed by one‐way ANOVA with multiple comparisons followed by Tukey's post test (ZIKV + treated vs. ZIKV + DMSO, where **p < 0.01 and ****p < 0.0001. AH‐D (100 µM) was employed as the positive control and DMSO as the negative control.

In subsequent experiments, the antiviral activity of the samples was evaluated in Vero CCL‐81 and SH‐SY5Y cells infected with ZIKV. The *T. glabrescens* extract exhibited a concentration‐dependent reduction in ZIKV load in Vero CCL‐81 cells, resulting in a 2.6‐log reduction at 30 µg/mL compared to the control group (ZIKV + DMSO) (Figure 2C). Importantly, this reduction in viral load did not correlate with any decrease in cell viability among the infected and treated cells when compared to the control group (ZIKV + DMSO) (Figure 2A,B). Similarly, the extract induced a significant 3.2‐log reduction in viral load in SH‐SY5Y cells at the highest tested concentration (Figure 2F). Notably, the cell viability of infected and treated SH‐SY5Y cells remained unaffected compared to the control group (ZIKV + DMSO) (Figure 2D,E). These findings collectively underscore the anti‐ZIKV effect of the *T. glabrescens* extract on both Vero CCL‐81 and SH‐SY5Y cells infected with the virus.

**FIGURE 3:**
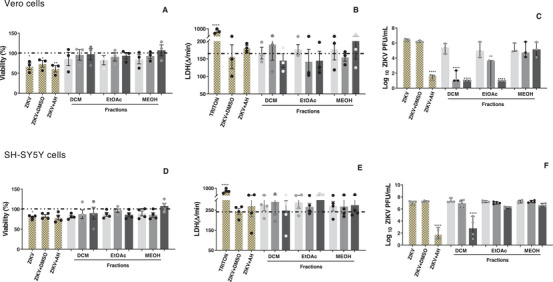
Effect of *Terminalia glabrescens* fractions on viral load and cell viability in ZIKV‐infected Vero CCL‐81 and SH‐SY5Y cells. Cell viability was assessed by the MTT (A, D) and LDH (B, E) assays. Fractions were tested at 3 µg/mL (light gray bars), 10 µg/mL (medium gray bars), and 30 µg/mL (black bars). Bars represent mean values ± SEM (A, B, D, E), where *p < 0.05; **p < 0.01 and ****p < 0.0001) versus the cell control group. Triton was used as the positive control for LDH assay. The dashed line (A, B, D, E) indicates the cell control (without extract and ZIKV). Viral load was determined by plaque assay (C, F), and data are expressed as log plaque‐forming units per milliliter (PFU/mL). Bars represent median values, and error bars indicate the 95% confidence intervals (C, F). Statistical analyses were performed by one‐way ANOVA with multiple comparisons followed by Tukey's post test (ZIKV + treated vs. ZIKV + DMSO, where **p < 0.01 and ****p < 0.0001. AH‐D (100 µM) was employed as the positive control and DMSO as the negative control.

The *T. glabrescens* fractions were tested in ZIKV‐infected Vero CCL‐81 and SH‐SY5Y cells at 3, 10, and 30 µg/mL. Notably, the DCM (0.9; 4.7 and 5.1 log reduction, respectively) and EtOAc (1.1; 3.7 and 5.1 log reduction, respectively) fractions promoted a concentration‐dependent reduction in viral load in Vero cells (Figure 3C). In SH‐SY5Y cells, the DCM fraction induced a substantial 4.0‐log reduction in viral load at the highest tested concentration (Figure 3F). Importantly, the viability of infected and treated cells was unaltered compared to the control group (ZIKV + DMSO) in both cell lines (Figure 3). Thus, the observed reduction in viral load suggests a clear anti‐ZIKV effect of the tested fractions in both Vero CCL‐81 and SH‐SY5Y cells.

The DCM and EtOAc fractions demonstrated activity against ZIKV‐infected Vero CCL‐81 cells, whereas only the DCM fraction exhibited activity against ZIKV in SH‐SY5Y cells. Notably, SH‐SY5Y cells are more sensitive and susceptible to ZIKV infection compared to Vero CCL‐81 cells, likely due to their tropism for neuronal cells [41]. This characteristic may contribute to a more efficient viral infection in SH‐SY5Y cells, potentially making it more challenging for the fractions to reverse or minimize the infection caused by ZIKV.

**FIGURE 4:**
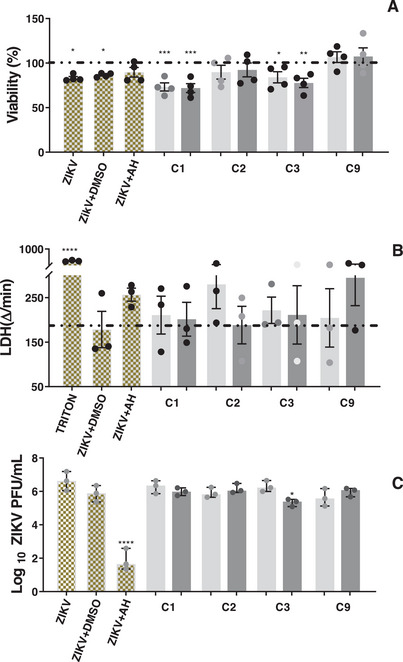
Effect of compounds and mixtures obtained from the DCM fraction of *Terminalia glabrescens* on viral load and cell viability in ZIKV‐infected SH‐SY5Y cells. Cell viability was evaluated by the MTT (A) and LDH (B) assays. Solids C1 (glutinol), C2 (a mixture of α‐ and β‐amyrin), C3 (β‐sitosterol), and C9 (a mixture of esters and friedelan‐3‐one) were tested at 10 µM (light gray bars) and 30 µM (medium gray bars). Triton was used as the positive control for LDH assay. The dashed line indicates the cell control (without extract and ZIKV). Bars represent mean values ± SEM (A, B), where *p < 0.05, **p < 0.01, and ***p < 0.001 versus the cell control group. Viral load was determined by plaque assay (C) and data are expressed as log plaque‐forming units per milliliter (PFU/mL). Bars represent median values, and error bars indicate the 95% confidence intervals (C). Statistical analyses were performed by one‐way ANOVA with multiple comparisons followed by Tukey's post test (ZIKV + treated vs. ZIKV+DMSO, where *p < 0.05 and ****p < 0.0001). AH‐D (100 µM) was employed as the positive control and DMSO as the negative control.

Based on these findings, the DCM fraction underwent further fractionation aimed at isolating the active compounds. The fractionation process yielded glutinol (C1), a mixture of α‐ and β‐amyrin (C2), β‐sitosterol (C3), and a mixture of esters and friedelan‐3‐one (C9) (see Figure 1 for chemical structures). These compounds/mixtures demonstrated no cytotoxicity to Vero CCL‐81 and SH‐SY5Y cells at concentrations up to 30 µM/30 µg/mL, as evidenced by the MTT and LDH assays (data not shown). Subsequently, the anti‐ZIKV activity of these compounds/mixtures was evaluated in ZIKV‐infected SH‐SY5Y cells. Only β‐sitosterol exhibited antiviral effects, demonstrating a reduction of 0.9 log in viral load at 30 µM (Figure 4C), while cell viability remained unaltered (Figure 4A,B).

Next, the median cytotoxic concentration (CC_50_), median effective concentration (EC_50_) and SI of the extract and β‐sitosterol were determined in ZIKV‐infected SH‐SY5Y cells. The *T. glabrescens* extract showed CC_50_ = 130.2 ± 27.5 µg/mL, EC_50_ = 20.4 ± 10.2 µg/mL, and SI = 6.4, while β‐sitosterol presented CC_50_ ≥ 300 µM, EC_50_ = 71.3 ± 7.1 µM and SI > 4.2. Both the extract and β‐sitosterol exhibited SIs above 4, a value that indicates promising candidates for future development of antiviral agents [42]. It should be noted that the extract exhibited a greater antiviral effect than β‐sitosterol (EC_50_ values of 20.4 ± 10.2 µg/mL and 71.3 ± 7.1 µM, respectively). This finding may suggest the presence of bioactive compounds other than β‐sitosterol and/or a synergistic effect between β‐sitosterol with a non‐identified constituent.

This study provides the first evidence of the anti‐ZIKV activity of β‐sitosterol. Interestingly, an in silico screening of 193 natural products through molecular docking using AutoDock Vina against the NS5 methyltransferase (MTase; PDB ID: 5WXB) and RNA‐dependent RNA polymerase (RdRp; PDB ID: 5U04) domains of the ZIKV identified β‐sitosterol as the compound with the most favorable binding energy [43]. The in vitro anti‐ZIKV activity demonstrated here corroborates these in silico findings. Moreover, β‐sitosterol has been reported to exert a strong inhibitory effect on the Na⁺/K⁺‐ATPase enzyme [44]. Notably, Na⁺/K⁺‐ATPase inhibitors such as ouabain and digoxin have been shown to reduce ZIKV infection in vitro during the replication stage and to lower viral load in vivo [45]. Therefore, it is feasible to suppose that the anti‐ZIKV effect of β‐sitosterol observed in infected SH‐SY5Y cells may involve a similar mechanism. Furthermore, this phytosterol has exhibited antiviral activity against HSV‐2 [46] and influenza A virus [47]. Collectively, these findings highlight β‐sitosterol as a promising broad‐spectrum antiviral candidate.

Another noteworthy aspect is the low toxicity of β‐sitosterol. Phytosterols, including β‐sitosterol, are abundant in plants and are typically consumed by humans at levels of 200–400 mg per day [48]. In this study, we determined a SI greater than 4.2 for β‐sitosterol in the SH‐SY5Y neuroblastoma cell line. The exact SI value could not be established, as the highest tested concentration (300 µM) did not induce 50% cell death (CC_50_ > 300 µM). These findings, therefore, reinforce the low cytotoxicity of this phytosterol. Although considered a safe compound, its high lipophilicity results in low systemic bioavailability, which limits its pharmacological efficacy in vivo unless optimized formulation strategies are employed, such as lipid‐based carriers, nanoemulsions, or inclusion complexes.

Fractionation of the ZIKV‐inhibiting DCM fraction also yielded glutinol and a mixture of α/β‐amyrin; however, neither compound reduced ZIKV plaque formation (Figure 4C). Nonetheless, glutinol may indirectly contribute to the antiviral activity of the DCM fraction through its inhibitory effects on pro‐inflammatory mediators in neuronal cells. Glutinol has been reported to exert neuroprotective effects by attenuating ethanol‐induced overexpression of TNF, caspase‐3, and PARP‐1 in the brain of neonatal mice [49]. Similarly, the anti‐inflammatory potential of α/β‐amyrin is well documented in several models. For instance, β‐amyrin significantly inhibited the release of IL‐1β, IL‐6, and TNF in LPS‐stimulated THP‐1 cells [50]. Therefore, α/β‐amyrin may also indirectly contribute to the antiviral effect of the DCM fraction by mitigating the release of inflammatory mediators in infected cells.

In summary, the ethanol extract of *T. glabrescens* leaves demonstrated anti‐ZIKV activity, with its fractionation yielding DCM and EtOAc fractions that exhibited antiviral properties, with the DCM fraction being more potent. Our phytochemical investigation revealed that the DCM fraction contains triterpenes and steroids, while the EtOAc fraction is rich in polyphenols. Among the DCM compounds tested, only β‐sitosterol showed significant anti‐ZIKV activity. To the best of our knowledge, this is the first report on anti‐ZIKV activity in *T. glabrescens*. Other species of *Terminalia* have been documented to possess antiviral properties, including *T. catappa* extracts and constituents against the human immunodeficiency virus [51], *T. chebula* against hepatitis B virus [52], and *T. arjuna* against herpes simplex virus type 2 (HSV‐2) [53].

#### 3.2.2 β‐Sitosterol Is Structurally Dissimilar to Known ZIKV Inhibitors

**FIGURE 5:**
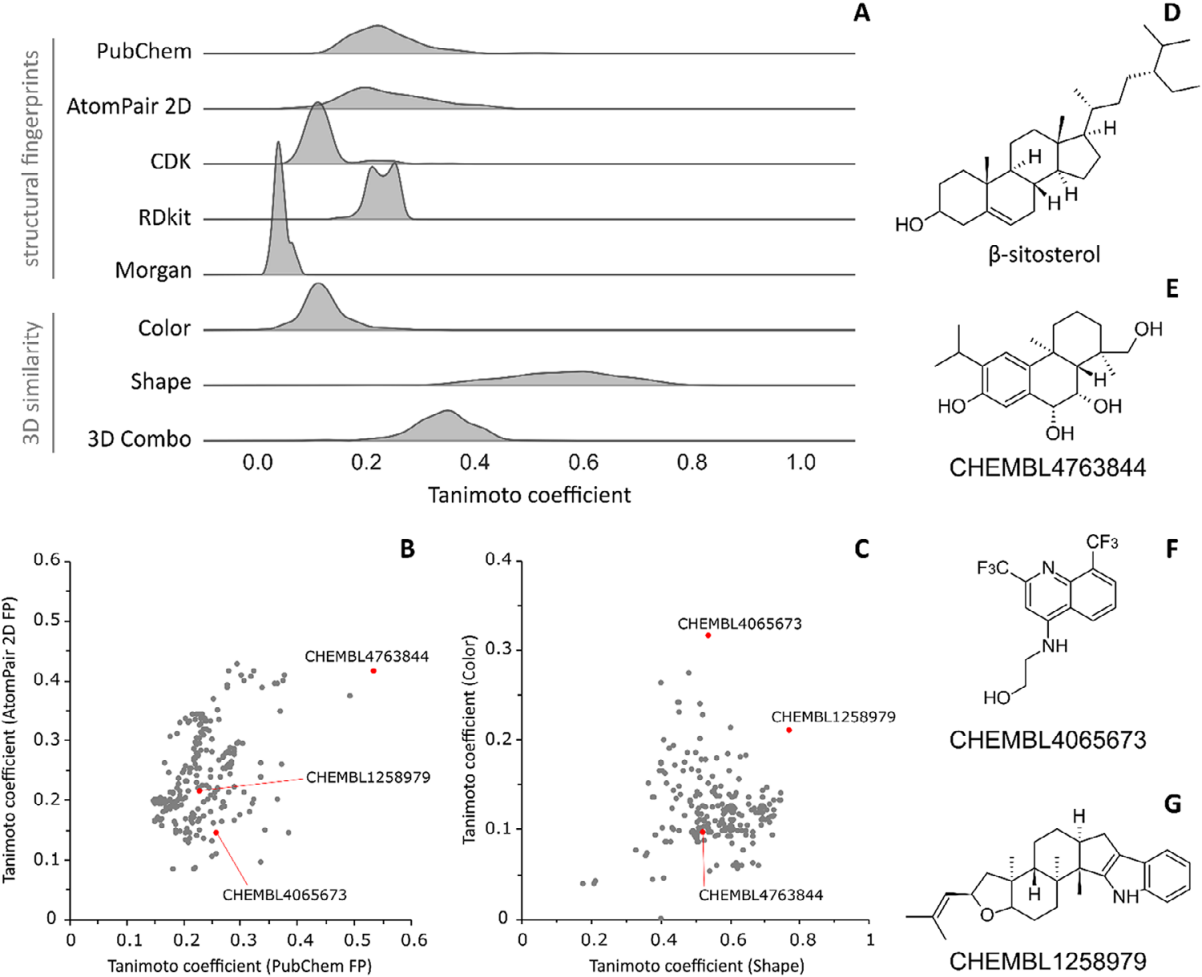
Similarity analysis of β‐sitosterol and ChEMBL active compounds against ZIKV and related molecular targets. Distribution of Tanimoto coefficients calculated with five different molecular fingerprints, pharmacophoric features (color), molecular shape, and 3D combo (color and shape) (A). Scatter plot of Tanimoto coefficients calculated with the two fingerprints which showed highest similarity coefficients with β‐sitosterol (PubChem FP and AtomPair 2D FP) (B), as well as three‐dimensional features (shape and color) (C). Chemical structures of β‐sitosterol (D) and the most similar compounds CHEMBL4763844 (E), CHEMBL4065673 (F), and CHEMBL1258979 (G).

The calculation of 2D similarity using structural fingerprints indicated that β‐sitosterol is not similar to compounds retrieved from ChEMBL with known anti‐ZIKV activities (Figure 5A). Tanimoto coefficients (Tc) distribution for all used fingerprints were not higher than 0.5, indicating that compounds share less than 50% of similarity. The most similar compound identified from ChEMBL is CHEMBL4763844 which showed Tc values equal to 0.53 and 0.42 for PubChem and AtomPair 2D fingerprints, respectively (Figure 5B). The two most similar compounds from 3D analysis were CHEMBL1258979 and CHEMBL4065673 with Tc values for shape comparison equal to 0.77 and 0.53 (Figure 5C), respectively, indicating that those compounds could fit into the same protein binding site. However, the color similarity of both in comparison with β‐sitosterol is lower than 0.4 suggesting that the three compounds were unlikely to form the same binding interactions with the same biological target.

All three structurally similar compounds reported in the literature lack a defined mechanism of action against ZIKV [54, 55, 56]. Therefore, the applied similarity‐based strategy was not informative. On the other hand, these results suggest that β‐sitosterol is a structurally unique compound, reinforcing the innovative potential of natural product profiling for the discovery of novel anti‐ZIKV agents. It should be noted, however, that the present similarity analysis has two main limitations. First, no experimental data are available for the compared compounds. Second, structurally dissimilar molecules may share a mechanism of action, whereas structurally similar ones may act through distinct mechanisms. To mitigate this limitation, we employed different similarity approaches, as multiple layers of molecular similarity can be considered [57]. Furthermore, our assumption is supported by previous in silico studies from our group, which indicate that structurally dissimilar compounds within a set of known inhibitors of a given target may suggest alternative mechanisms of action or interactions with distinct binding sites [25, 58].

## 4 Conclusions

The findings demonstrate that *T. glabrescens* leaves contain bioactive constituents with potent anti‐ZIKV activity, particularly within the DCM fraction. Chemical characterization of the ethanol extract by LC‐MS led to the identification of 27 phenolic compounds, whereas GC–MS analyses of the DCM fraction revealed esters, hydrocarbons, an alcohol, and a triterpene. The isolation and identification of β‐sitosterol as the major active compound highlights its potential as a promising lead for antiviral drug development, as it effectively inhibited ZIKV replication in both Vero and SH‐SY5Y cells with low cytotoxicity. Furthermore, the structural dissimilarity of β‐sitosterol from known anti‐ZIKV compounds underscores its novelty and potential to serve as a template for new antiviral derivative. Overall, this study provides the first evidence supporting *T. glabrescens* and β‐sitosterol as valuable sources for the development of therapeutic candidates against ZIKV infection.

## Conflicts of Interest

The authors declare no conflicts of interest.

## Supporting information




**Supporting File 1**: cbdv70824‐sup‐0001‐SuppMat.pdf

## Data Availability

The data that support the findings of this study are available from the corresponding author upon reasonable request.
