# Supplementary material for: Bioactivity‐Guided Isolation of β‐Sitosterol From Terminalia glabrescens as a Potent Anti‐Zika Virus Agent
Source: Chem Biodivers. 2026 Jan 14;23(1):e02860. doi: 10.1002/cbdv.202502860 (PMC12802818; doi:10.1002/cbdv.202502860)
Supplement: Supplementary file 1 — Supporting File 1: cbdv70824‐sup‐0001‐SuppMat.pdf [file CBDV-23-e02860-s001.pdf]

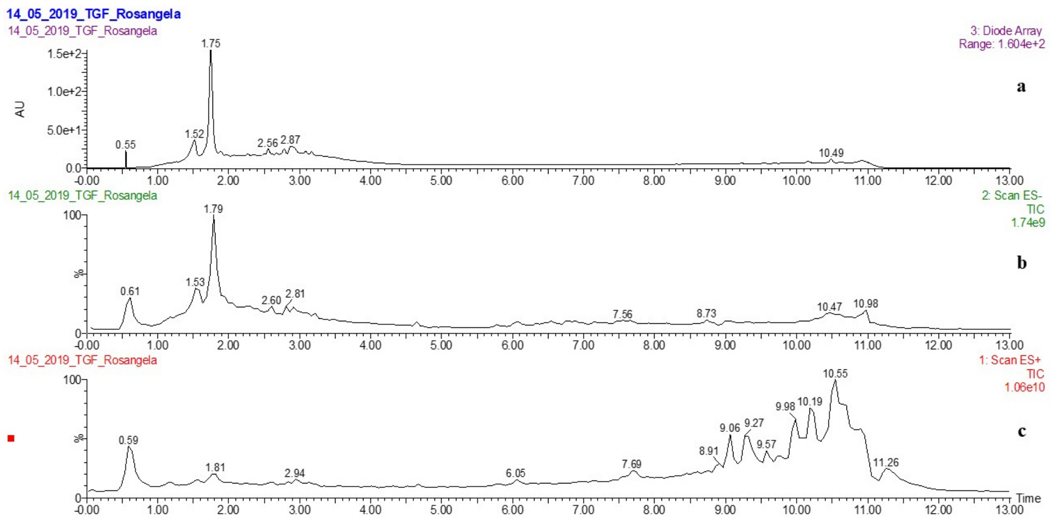

Figure S1. Chromatograms obtained by UPLC-DAD-ESI-MS/MS for the ethanolic extract (EE) of *T. glabrescens* leaves in scan mode with UV detection (a), and in ESI^{-} (b) and ESI^{+} (c) modes in the exploratory run. Chromatographic and MS conditions: see Experimental Section.

Figures S2–S4 illustrate an example of the putative identification of compounds by UPLC-DAD-ESI-MS/MS in the ethanol extract (EE) of *T. glabrescens* leaves and the corresponding ethyl acetate (EtOAc) and methanol (MeOH) fractions, as described in Table 1 of the manuscript.

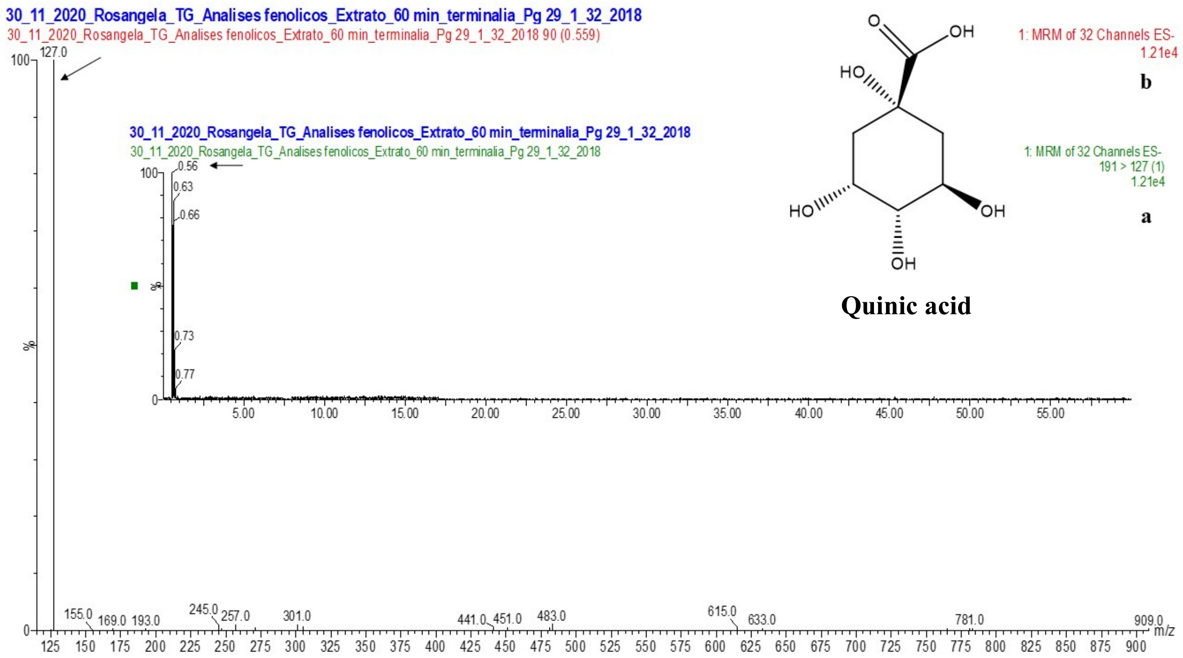

Figure S2. MRM spectrum for the transition *m/z* 191 → 127 (a) and MS/MS mass spectrum for the ion at *m/z* 191 Da (b) obtained by UPLC-DAD-ESI-MS/MS for the ethanolic extract of *T. glabrescens* leaves acquired in the negative ionization mode (ESI⁻). MS conditions: see Experimental Section.

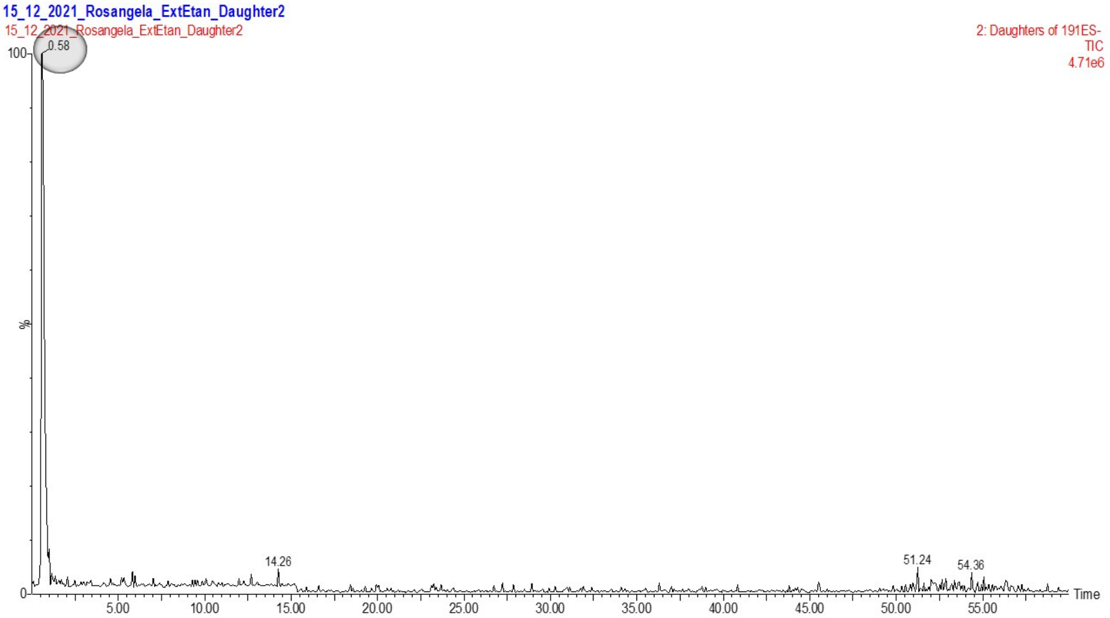

Figure S3. Chromatogram obtained in the daughter scan experiment (ESI$^-$) for the ion at *m/z* 191 Da by UPLC-DAD-ESI-MS/MS for the ethanolic extract (EE) of *T. glabrescens* leaves. Chromatographic and MS conditions: see Experimental Section.

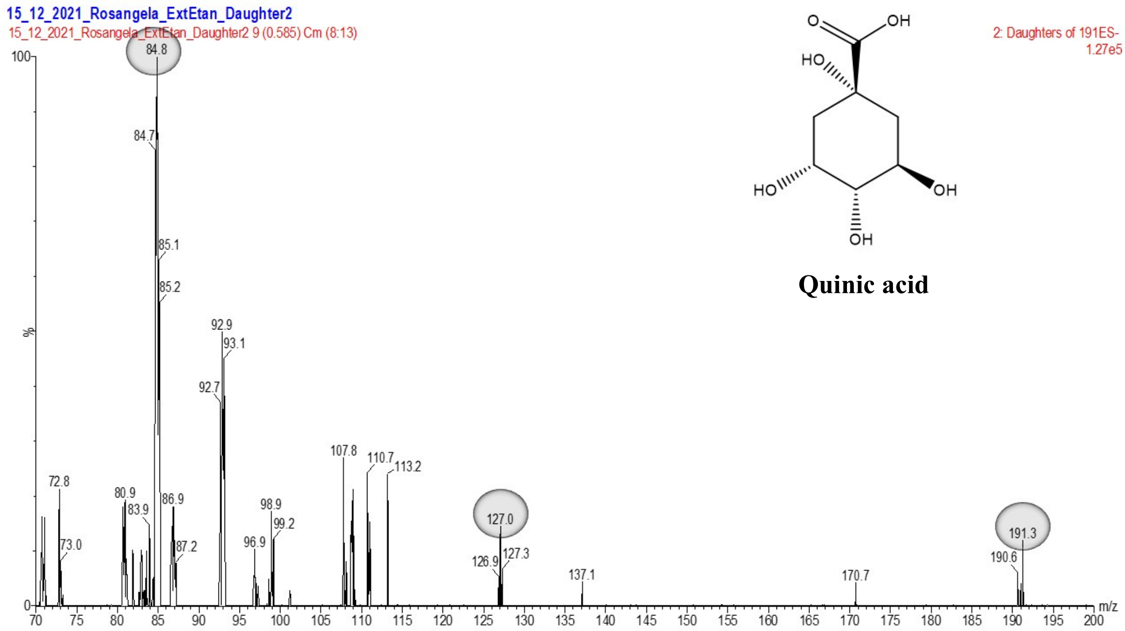

Figure S4. Spectrum obtained by UPLC-DAD-ESI-MS/MS in the daughter scan experiment (ESI^{-}) for the ion at *m/z* 191 Da, with the corresponding fragment ions that enabled the putative identification of the highlighted compound in the ethanolic extract (EE) of *T. glabrescens* leaves. Chromatographic and MS conditions: see Experimental Section.

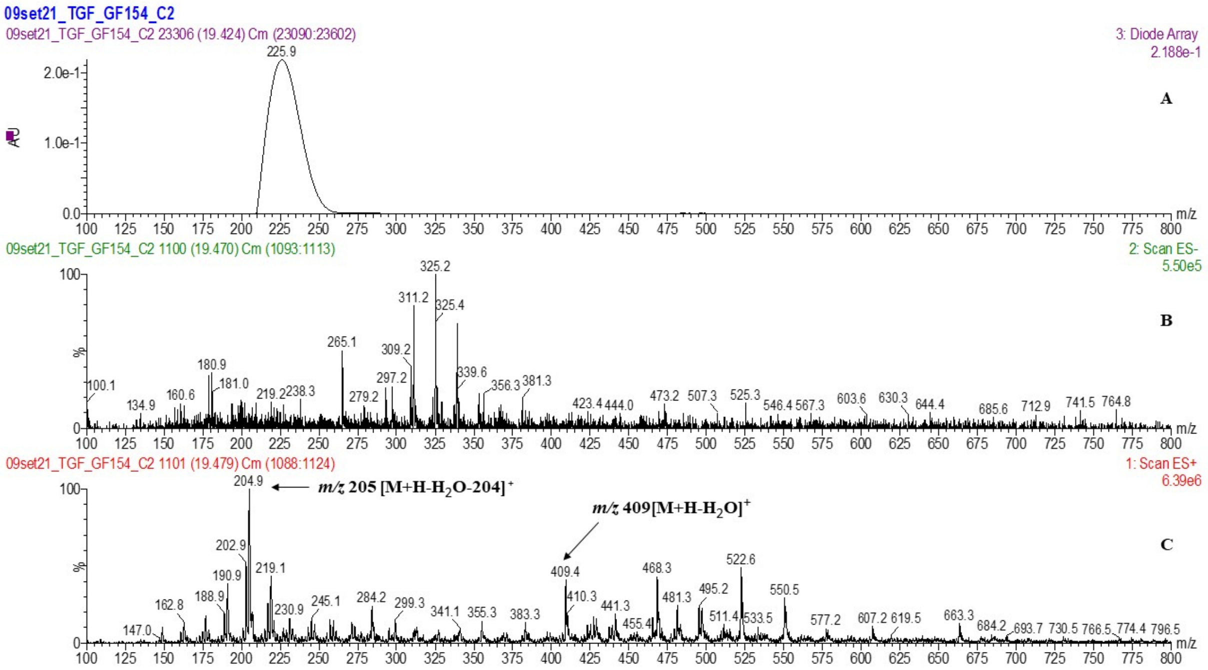

Figure S5. UV spectrum (A) and mass spectra obtained for C1 (glutinol) by UPLC-DAD-ESI-MS/MS in positive ionization mode ESI$^+$ (B) and negative ionization mode ESI$^-$ (C). Chromatographic and spectroscopic conditions: see Experimental Section.

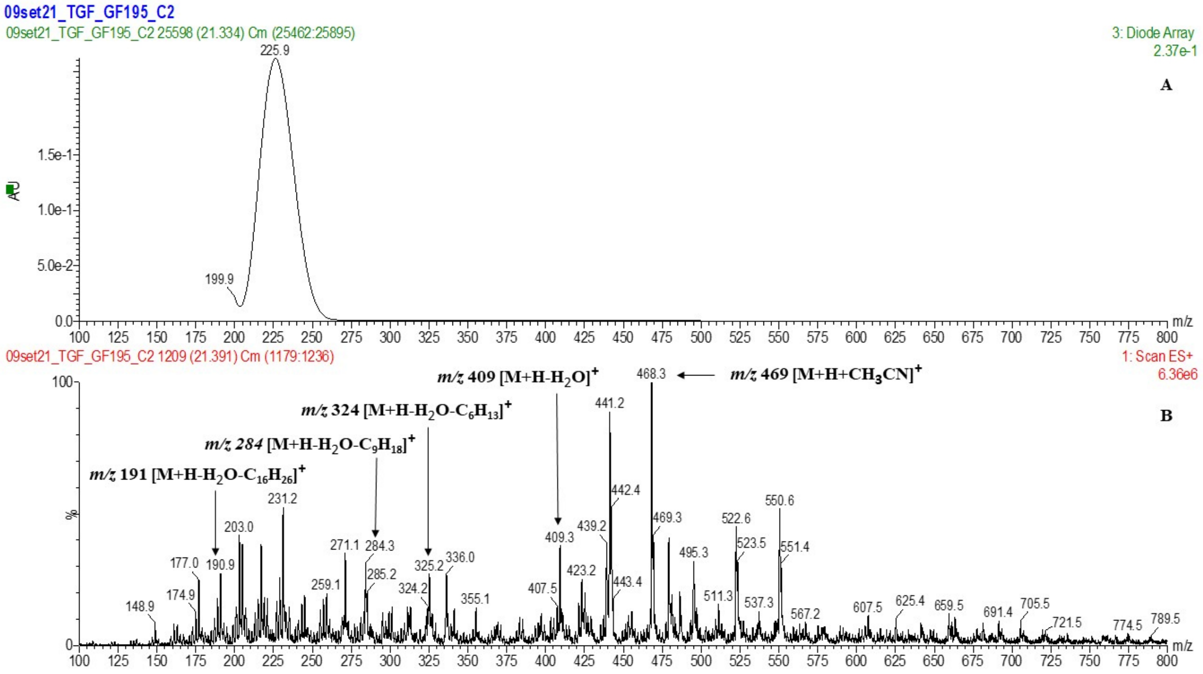

Figure S6. UV spectrum (A) and mass spectrum obtained for C2 (α/β-amyrin) by UPLC-DAD-ESI-MS/MS in negative ionization mode ESI⁻ (B). Chromatographic and spectroscopic conditions: see Experimental Section.

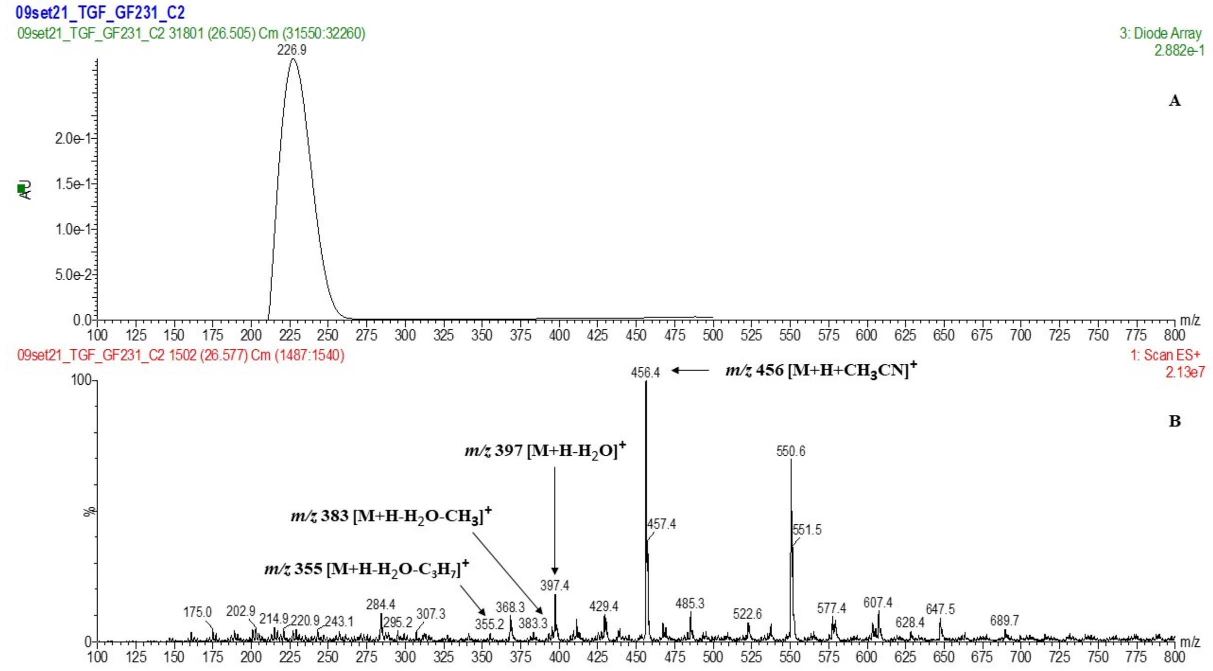

Figure S7. UV spectrum (A) and mass spectrum obtained for C3 (β-sitosterol) by UPLC-DAD-ESI-MS/MS in positive ionization mode ESI^+ (B). Chromatographic and spectroscopic conditions: see Experimental Section.
